# Using a systematic and quantitative approach to generate new insights into drug loading of PLGA nanoparticles using nanoprecipitation†

**DOI:** 10.1039/d4na00087k

**Published:** 2024-05-08

**Authors:** Sherif I. Hamdallah, Randa Zoqlam, Bin Yang, Andrew Campbell, Rebecca Booth, Jonathan Booth, Peter Belton, Sheng Qi

**Affiliations:** a School of Pharmacy, University of East Anglia Norwich NR4 7TJ UK S.hamdallah@uea.ac.uk Sheng.qi@uea.ac.uk; b Department of Pharmaceutics, Faculty of Pharmacy, Alexandria University Alexandria Egypt; c School of Pharmacy, University College London London WC1N 1AX UK r.zoqlam@ucl.ac.uk; d Advanced Drug Delivery, Pharmaceutical Sciences, The Discovery Center (DISC) 1 Francis Crick Avenue Cambridge CB2 0AA UK bin.yang4@astrazeneca.com Andrew.Campbell@astrazeneca.com; e New Modalities and Parenteral Development, Pharmaceutical Technology & Development, Operations, AstraZeneca Macclesfield SK10 2NA UK Rebecca.J.Booth@astrazeneca.com Jonathan.Booth@astrazeneca.com; f School of Chemistry, University of East Anglia Norwich NR4 7TJ UK p.belton@uea.ac.uk

## Abstract

The synthesis of drug-loaded PLGA nanoparticles through nanoprecipitation in solvent/antisolvent mixtures is well reported but lacks clarity in explaining drug loading mechanisms and the prediction of efficiency of drug entrapment. Various methods using physical parameters such as log *P* and solid-state drug-polymer solubility aim to predict the intensity of drug–polymer interactions but lack precision. In particular, the zero-enthalpy method for drug/polymer solubility may be intrinsically inaccurate, as we demonstrate. Conventional measurement of loading capacity (LC), expressed in weight ratios, can be misleading for comparing different drugs and we stress the importance of using molar units. This research aims to provide new insights and critically evaluate the established methodologies for drug loading of PLGA nanoparticles. The study employs four model drugs with varying solubilities in solvent/antisolvent mixtures, log *P* values, and solid-state solubility in PLGA: ketoprofen (KPN), indomethacin (IND), sorafenib (SFN), and clofazimine (CFZ). This study highlights that drug loading efficiency is primarily influenced by the drug's solubilities within the solvent system. We emphasise that both kinetic and thermodynamic factors play a role in the behaviour of the system by considering the changes in drug solubility during mixing. The study introduces a pseudo-constant K* to characterise drug–polymer interactions, with CFZ and SFN showing the highest K* values. Interestingly, while IND and KPN have lower K* values, they achieve higher loading capacities due to their greater solubilities, indicating the key role of solubility in determining LC.

## Abstract 2

An exploratory investigation into the molecular-level determinants influencing efficient drug loading in PLGA nanoparticles using a nanoprecipitation method.

## Graphical abstract

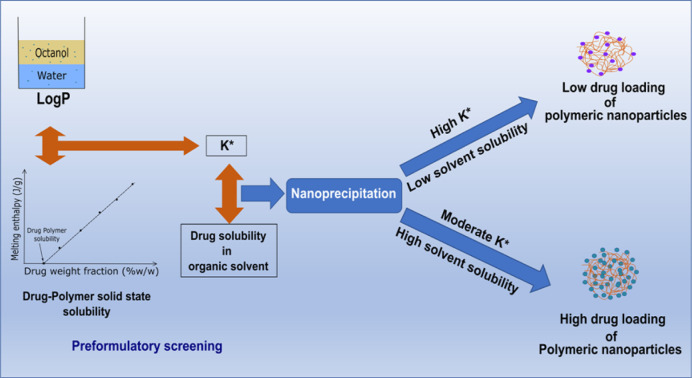

## 1. Introduction

Poly(lactic-*co*-glycolic) acid (PLGA) nanoparticles fabricated by nanoprecipitation have shown considerable potential in the pharmaceutical drug delivery field. This is attributed to a group of appealing properties, including their biodegradability, and biocompatibility, besides providing a sustained drug release profile and optimal drug bioavailability.^1,2^ Nanoprecipitation, also known as interfacial deposition or solvent displacement, is one of the most adopted techniques for nanoparticle (NP) fabrication owing to its simplicity, good reproducibility, ease of scalability, and feasibility of producing small NPs of submicron size with a narrow size distribution profile.^3,4^ Precipitation or phase separation of the required components (polymer/drug) from a solvent system is considered the typical process for NP fabrication using this approach.^5–7^ Whilst phase separation can be induced by any physical change in the solvent–antisolvent system, such as temperature, pH, or any change in the solubility of the components.^3,4,8,9^ we have selected the commonly used solvent/antisolvent system to explore the role of drug solubility and PLGA supersaturation on the ability of a drug to be entrapped by the nanoparticles.

Fabrication of drug-loaded PLGA NPs using this nanoprecipitation method requires dissolving the PLGA and drug in a water-miscible organic solvent and then thoroughly mixing it with an aqueous antisolvent (water/aqueous buffer) to achieve the supersaturated state and induce PLGA precipitation.^3,6,10^ Depending on the drug's degree of supersaturation in the solvent/antisolvent mixture, drug precipitation may or may not occur. Therefore, the degree of supersaturation (DOS), defined as the ratio between the component concentration in the solvent–antisolvent mixture after mixing and its equilibrium solubility in that mixture, is an important factor in the process. The degree of supersaturation can affect the mechanism of drug entrapment. For example, a high degree of supersaturation shortens the precipitation time and may cause the precipitation to start before homogenous mixing is achieved.^7,11^ This can reduce the drug entrapment by forming two phases of PLGA NPs and drug particles and may have consequences for accurately determining drug loading if drug particles are not completely separated from the nanoparticles during analysis.

Since the properties of the NPs depend on the parameters that induce and control the precipitation, many studies have investigated the effect of processing parameters, such as the polymer concentration, solvent–antisolvent volume ratio, solvent–antisolvent miscibility and mixing efficiency on the properties of the blank PLGA NPs.^6,10,12–14^ However, fewer studies have examined the impact of these parameters on drug loading.^15–18^ In addition, the drug entrapment by PLGA NPs has tended to be treated in the literature on a descriptive basis by demonstrating the impact of each processing parameter on a specific drug and application. However, there is a lack of generalised conclusions regarding the impact of the processing parameters on the drug entrapment mechanism and efficiency. This highlights one of the problems of studies in this area; many variables are involved but, in the literature, typically two parameters are commonly used to quantify the entrapment of drug by the polymer. These are the loading capacity (LC%) defined by:1
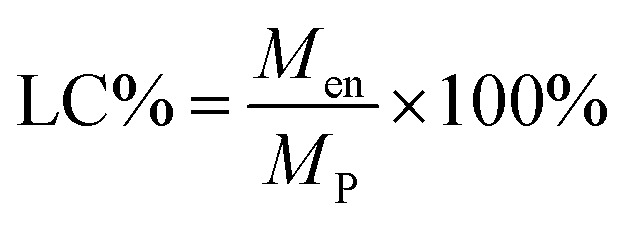
where *M*_en_ is the mass of drug entrapped by the nanoparticle and *M*_P_ is the total mass of the drug loaded nanoparticles.

The other parameter used is the entrapment/encapsulation efficiency (EE%) defined by:2
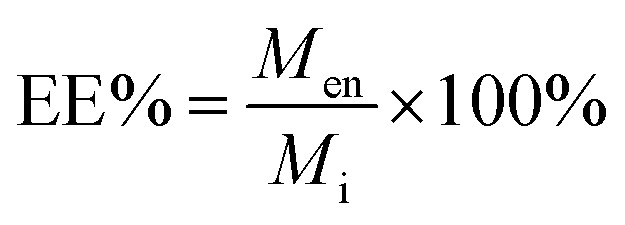
where *M*_i_ is the total initial mass of drug added to the system. Whilst loading capacity (LC%) in mass percentage is a useful guide in clinical applications it can be misleading when comparing the entrapment of different drugs by polymer. One drug with twice the molecular weight of another drug will show twice the loading capacity for the higher molecular weight drug, even though the nanoparticles entrap the same number of molecules of each drug.

EE% has the advantage that it is mass ratio and is therefore independent of molecular weight and can be used directly as measure of the degree of interaction of the drug and polymer. Drug entrapment assessment methods depend on the solubility of the unentrapped drug in the dispersion medium. For formulations in which the unentrapped drug is soluble in the dispersion medium, the ‘indirect’ method can be used by quantifying the unentrapped free drug from the supernatant.^19^ On the other hand, for those formulations in which the unentrapped drug is insoluble in the dispersion medium, ‘direct’ assay methods of the drug from nanoparticles should be adopted.^20,21^

Predicting the drug EE% by PLGA NPs prepared through nanoprecipitation based on its hydrophobicity (log *P*) is widely adopted in the literature.^17,22,23^ This is related to the partitioning effect, whereby hydrophilic drug molecules preferentially diffuse from the polymeric organic phase into the external water phase, resulting in a low EE%. Although widely accepted, this suggests that a drug with a higher log *P* value (>3.5) will always show a higher EE% compared to a drug with a lower log *P* value, as sufficient supersaturation can be easily achieved during antisolvent mixing resulting in high nucleation rates,^24,25^ however this is not necessarily the case.^26–29^ Treating drugs simply in terms of their hydrophilicity or hydrophobicity, underestimates the system's complexity. Adopting this assumption ignores the impact of other factors, such as the solubility of the drug in the solvent–antisolvent mixture and whether the drug is molecularly dispersed or suspended as solid particles during the mixing process. Other approaches relate the EE% to the drug's solubility in PLGA. These rely on using thermal analysis methods, such as the melting point depression method, the recrystallization method, the annealing method, and the zero-enthalpy extrapolation method, to measure the solid state drug solubility in PLGA.^30–33^ Therefore, it is not clear how they might directly predict the drug loading during the nanoprecipitation process in a liquid medium, where a solvent and anti-solvent are used for NPs synthesis.

**Fig. 1:**
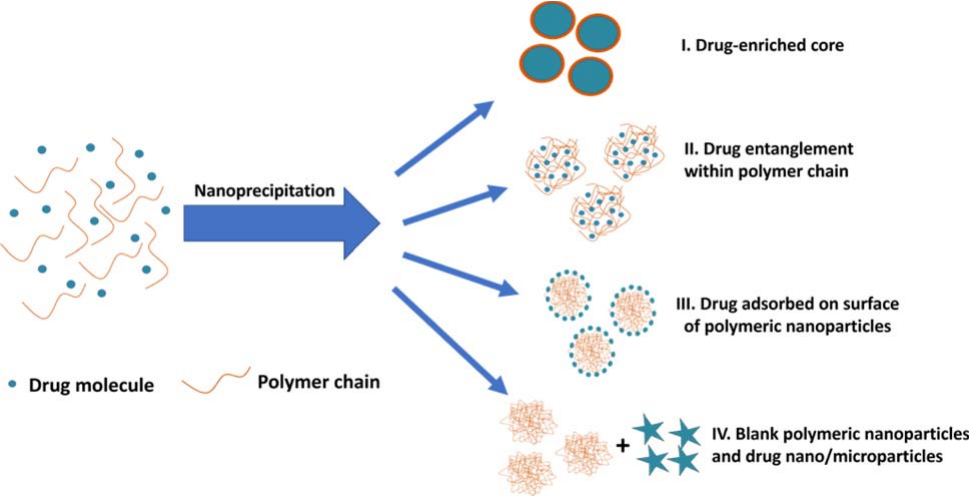
Different possible mechanisms of drug entrapment into polymeric nanoparticles during nanoprecipitation process.

During drug entrapment/encapsulation, a number of mechanisms may come into play (Fig. 1): precipitation of the drug may occur more rapidly than the polymer, leading to the formation of drug-enriched core polymeric nanoparticles (Fig. 1(I)); alternatively, drug molecules can become entrapped within particles through entanglement during nanoparticle synthesis (Fig. 1(II)).^34–36^ Additionally, drug molecules may adsorb onto the surface of nanoparticles (Fig. 1(III)).^37–39^ A further possibility is that drug is absorbed into the main body of the nanoparticle by diffusion after the nanoparticles are formed. However, there is little support for this view in the literature and molecular dynamics simulations suggest that drug is more likely to be adsorbed on the surface of the particle.^16,17,40^ Conversely, drug and polymer may precipitate independently if their precipitation kinetics differ resulting in the formation of blank polymeric nanoparticles and drug nano/microparticles (Fig. 1(IV)).

In many studies only one drug is used and the degree of supersaturation of several drugs are not systematically compared. Hence, the objective of this research is to offer new perspectives on the process of drug loading within PLGA NPs, achieved through a critical assessment of the conventional concepts and methodologies used to evaluate the efficacy of drug loading into PLGA NPs produced through nanoprecipitation. Consequently, this investigation seeks to elucidate the influence of nanoprecipitation process parameters on the efficiency of drug loading into PLGA NPs, with a particular emphasis on discerning the impact of drug and polymer solubility within the solvent–antisolvent mixture on the underlying mechanisms and efficiency of drug loading.

**Fig. 2:**
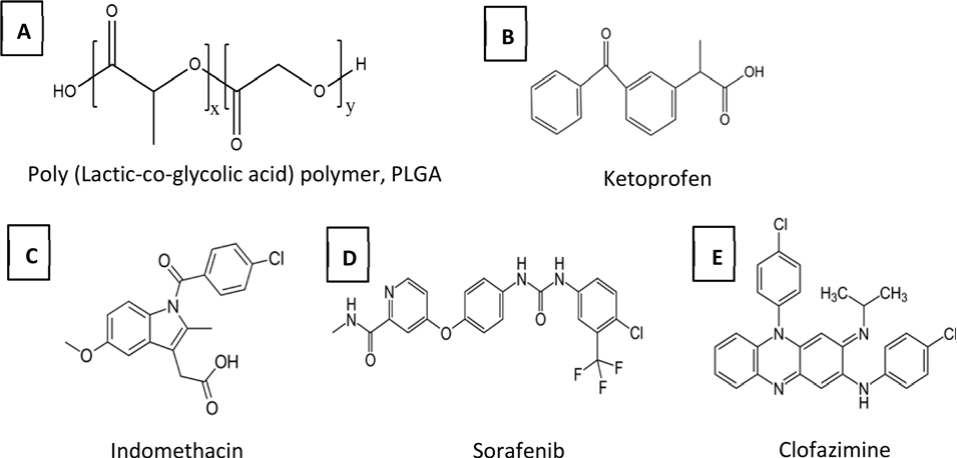
Chemical structures of PLGA polymer and the four model drugs.

In this study four model drugs (Fig. 2), ketoprofen (KPN), indomethacin (IND), sorafenib (SFN), and clofazimine (CFZ), representing a range of solubilities in the precipitating media, log *P* values and solid-state solubility in PLGA, are used. In order to keep the thermodynamic activity of the drugs constant, each is used at a concentration of 0.8 times of its solubility (0.8x DOS) in the solvent mixture used.^41^ In the case of KPN (where the solubility was high such that different levels of concentration were readily experimentally accessible) the effects of different levels of supersaturation were also explored.

## 2. Experimental section

### 2.1. Materials

Poly Lactic-*co*-Glycolic Acid (PLGA) 50 : 50 ester terminated (M. wt. 38 000–54 000 Da), IND, KPN, CFZ, acetonitrile (ACN, HPLC grade, purity ≥ 99.8%), methanol (HPLC grade, purity ≥ 99.9%), phosphoric acid (85%), acetic acid, triethylamine (TEA), and phosphate buffered saline (PBS) in tablet form were purchased from Sigma Aldrich (Poole, UK). SFN was purchased from LC Labs (Woburn, MA, USA). Milli-Q water (MQW) was obtained from Milli-Q systems (Millipore, Watford, UK) and used as antisolvent. The solvent/anti-solvent mixture was fixed to be 20% ACN/MQW.

### 2.2. Methods

#### 2.2.1. PLGA solubility study

To measure the degree of supersaturation (DOS) of PLGA during the solvent/anti-solvent mixing process, it is crucial to measure its solubility in the solvent/anti-solvent mixture. Therefore, the solubility of PLGA in 20% ACN/Water was measured using the gravimetric method.^1^ An excess mass of PLGA was placed into a vial with 1 mL of 20% v/v ACN/water. The vials were incubated in an IKA KS 3000 i-control shaking incubator (IKA®-Werke GmbH & Co. KG, Germany) at 100 rpm and 25 °C for 48 h followed by another 24 h without shaking for equilibration. Then, the supernatant was collected and filtered using a 0.22 μm syringe filter from which precise volumes (*V*) were transferred into pre-weighed vials (*m*_1_). The solvent was evaporated by heating in an oven at 60 °C for 6 h, and the vials were weighed again (*m*_2_). The solubility of the polymer was calculated using the following eqn (3):3
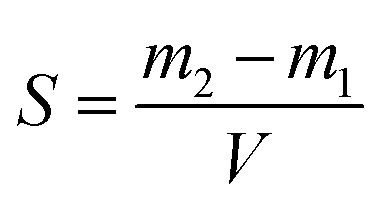


#### 2.2.2. Solubility of the model drugs in water and solvent/antisolvent mixture

To measure the DOS of the drugs during the solvent/anti-solvent mixing process and after solvent evaporation, it is crucial to measure the drugs solubility in the solvent/anti-solvent mixture and Milli-Q water. Therefore, drugs solubilities were measured by adding an excess mass of the drug into a vial with 2 mL of Milli-Q water or 20% v/v ACN/water. The vials were incubated in an IKA KS 3000 i-control shaking incubator (IKA^®^-Werke GmbH & Co. KG, Germany) at 100 rpm and 25 °C for 48 h followed by another 24 h without shaking for equilibration. Then, the supernatant was collected and filtered using a 0.45 μm syringe filter to be assayed on the HPLC using the methods shown in the following section.

#### 2.2.3. Drugs/PLGA solubility in solid state using differential scanning calorimetry (DSC)

A Discovery DSC2500 (TA Instruments, Delaware, United States) was used to quantify the solubility (w/w%) of different drugs in PLGA polymer in solid state using zero-enthalpy extrapolation method.^32,33^ Briefly, different drug masses (in 10% increments of drug weight fraction w/w%) were physically mixed with PLGA in hermitically sealed T-zero aluminium pan with a total mixture weight of 5 ± 0.2 mg. The DSC instrument was calibrated prior to sample measurements using pure indium. All samples were tested over a temperature range from 25 °C to a temperature higher than the melting point of the drug with a heating rate of 2 °C min^−1^. Nitrogen purge gas with a flow rate of 50 mL min^−1^ was used throughout the experiments. TA Trios software was used for the data analysis. All tests were performed in triplicates.

#### 2.2.4. Drug loading measurements of PLGA NPs

Different masses of IND, SFN and CFZ (masses that represent 0.8 times of their saturated solubility in a total of 5 mL of 20% ACN/Water) were dissolved in 1 mL of ACN with different masses of PLGA representing different DOSs in the solvent/anti-solvent mixture as shown in Table 1. KPN was used as a model drug to study the effect of the drug DOS on the Drug-PLGA interactions in the solvent/anti-solvent mixture.

**Table 1:** Masses of drugs/PLGA and the DOSs in the solvent/anti-solvent mixture used in this study

Drug/polymer	Solubility in solvent/antisolvent mixture (20% ACN) (μg mL^−1^)	Solubility in solvent/antisolvent mixture (20% ACN) (μmol mL^−1^)	DOS	Mass in 1 mL ACN (μg)
IND	142	0.40	0.8	568
KPN	3940	15.51	0.2	3940
			0.4	7880
			0.8	15 760
SFN	0.307	6.60 × 10^−4^	0.8	1.23
CFZ	0.167	3.51 × 10^−4^	0.8	0.668
PLGA	106	2.30 × 10^−3^	1.5	795
			5	2650
			10	5300
			15	7950
			20	10 600

Therefore, different masses of KPN (masses that represent 0.2, 0.4 and 0.8 times of its saturated solubility in a total of 5 mL of 20% ACN/Water) were dissolved in 1 mL of ACN containing PLGA as mentioned above. Then, acetonitrile solution containing the PLGA, and drug was dripped at a rate 1 mL min^−1^ into 4 mL of Milli-Q water (antisolvent) which was stirred at 300 rpm for 5 min (ESI, Fig. S1†).

Since the used drug concentrations are below solubility limit in 20% ACN/water mixture, entrapment efficiency (EE%) and loading capacity (LC%) were quantified using the indirect method. Drug content in the supernatant was measured after ultrafiltration using vivaspin^®^ (MWCO 100 kDa) at 8000 rpm for 10 min assuming that the amount of drug not in supernatant is encapsulated in PLGA NPs.

EE% and LC% were calculated using the following equations:4
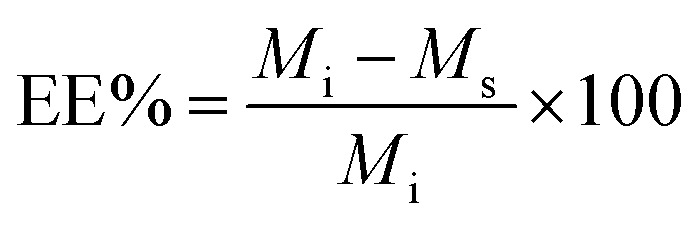
5
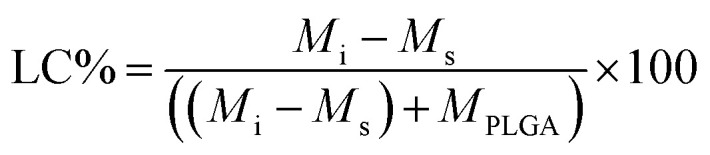
where, *M*_i_ is the initial mass of the API added to the system, *M*_s_ is the mass of the API in the supernatant, and *M*_PLGA_ is the total mass of PLGA used.

To investigate the input of surface adsorption as a possible mechanism of drug loading in PLGA NPs, KPN-loaded PLGA NPs were prepared through two-step process (ESI, Fig. S2†). First step was the preparation of blank PLGA NPs as previously elaborated using 5.3 mg of PLGA giving 10x DOS of PLGA NPs in 20% ACN. The second step was the addition of 5 mL of KPN solution in 20%ACN (at 0.8x or 0.4x DOS) and the dispersion was kept under stirring at 300 rpm for 5 min before drug loading assay. The final concentrations were 5x DOS of PLGA NPs with either 0.4x or 0.2x DOS of KPN.

#### 2.2.5. Physicochemical characterisation of drug loaded PLGA NPs

The NPs were characterised for their size and shape using dynamic light scattering (DLS) (Zetasizer Nano, Malvern Instruments Ltd, Malvern, UK) and transmission electron microscopy (TEM, JEOL JEM2010 200 kV, Japan) was used to analyse the morphologies of blank and drug-loaded NPs. 10 μL of each nanoparticle dispersion was placed on the grid for 1 min. The excess suspension was dried using filter paper before staining the grid with phosphotungstic acid (2%, pH 6.8) to enhance the contrast of the sample.

#### 2.2.6. HPLC assay methods

For drugs quantification, an Agilent 1260 affinity II (Agilent, UK) with autosampler and dual channel UV detector equipped with Agilent HC-C18 column (4.6 × 250 mm, 5 μm, 400 bar) was used. Different chromatographic conditions have been adopted for different drugs as shown in Table 2.

**Table 2:** Chromatographic conditions for drugs quantification

Drug	Mobile phase	Flow rate (mL min^−1^)	Detection wavelength (nm)	Ref.
KPN	Methanol (70%) and MQW (30%), pH at 3.3 using acetic acid	1	260	42
IND	Acetonitrile (63%) and MQW (37%) containing 0.2% phosphoric acid (pH at 2)	1.2	254	43
SFN	Acetonitrile (75%) and MQW (30%) containing 0.03% triethylamine	1	265	44
CFZ	Methanol (80%) and PBS (20%) at pH 4 by phosphoric acid	1	284	45

## 3. Results and discussion

### 3.1. Drugs/PLGA solid state solubility measured using DSC

The experimental determination of drug-polymer solubility at room temperature has some limitations related to the high viscosity of polymers and the solid nature of most drugs at ambient temperature. Therefore, several methods relying on computational modelling, such as PC-SAFT and thermal analysis protocols using the DSC at elevated temperatures, have been proposed in the literature to predict drug-polymer solubility.^30–33^

The melting point depression method, the recrystallization method, the annealing method, and the zero-enthalpy extrapolation method are among the most used methods.^46–48^ The zero-enthalpy extrapolation method relies on the assumption that the dissolved fraction of the drug in the polymeric matrix does not contribute to fusion enthalpy. Thus, the drug content at which there is no fusion enthalpy represents the drug solubility in the polymer.

Several studies suggest that the solid-state solubility of hydrophobic drugs in polymers could indicate their entrapment efficiency and release profile from the NPs.^33,39,49,50^

For instance, a study by Panyam *et al.* used dexamethasone and flutamide to study the correlation between the drug-polymer solid-state solubility and the drug entrapment efficiency.^33^ The study noted a positive correlation between the solid-state drug-polymer solubility and the drug entrapment efficiency but an inverse correlation with the cumulative drug release. However, a number of questions regarding the general validity of the method need to be addressed.

Firstly, the relevance of this measurement, in an essentially solvent-free environment, to what happens in a precipitating system where entrapment is involved, and the final material may not be at equilibrium. Secondly, the experimental method is itself somewhat suspect as it is predicated on the assumption that the polymer is saturated by drug during the heating process before any melting is observed. However, the apparent solubility of drugs in polymer has been shown to be dependent on the DSC scanning rate^30^ and does not take full account of the role of the heat of solution of the drug in polymer. There is also the possibility of dissolution of polymer in drug at very high drug to polymer ratios.^32^ (See the quantitative discussion provided in the ESI†). There may be some evidence of the role of polymer solubility in the molten drug in the CFZ data. Here, at the 90% drug level, a deviation from the linearity of the preceding points is seen (see ESI†). In general, the thermometric method can only reliably give a semi-quantitative indication of drug solubility and can provide an indication of solubility ranking when results from the same experimental set up are compared.

**Fig. 3:**
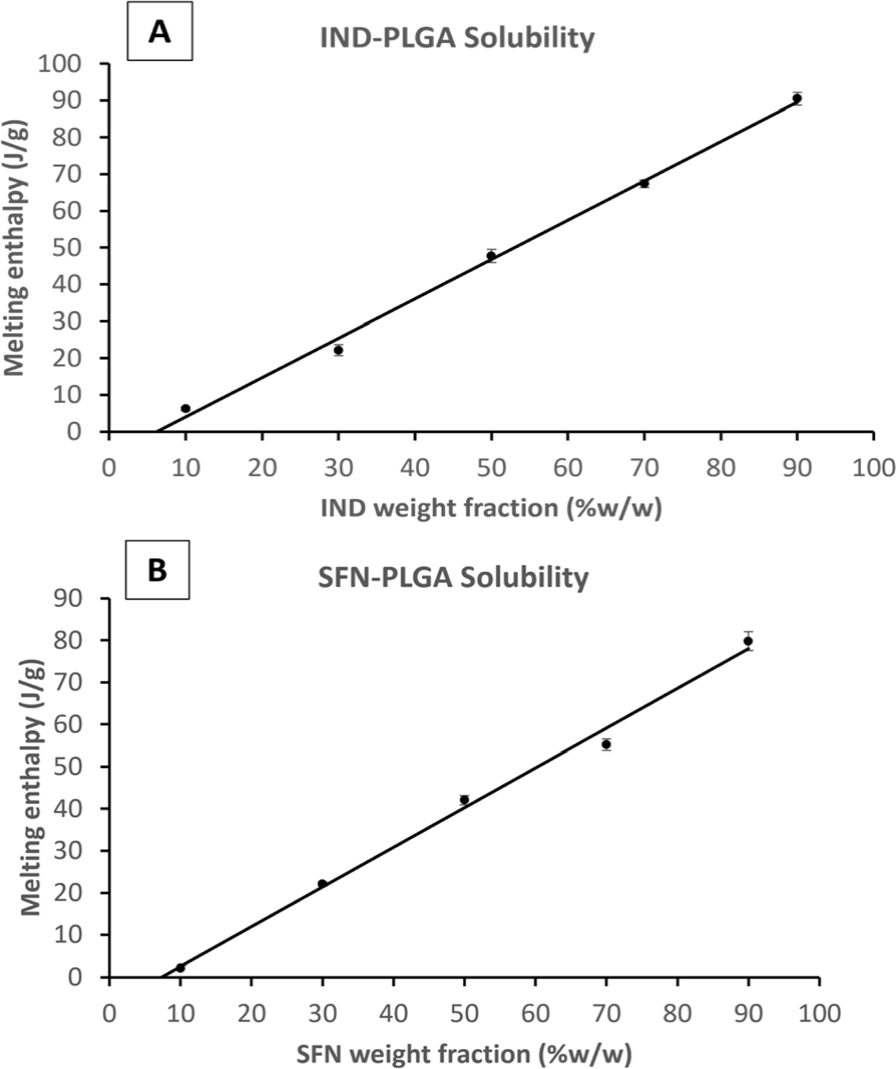
Plots of melting enthalpy of the physical mixtures of PLGA with (A) IND and (B) SFN.

To compare the measured drug-PLGA solid solubility with the final drug loading capacity, the enthalpy of the fusion (melting) peak of each drug-PLGA physical mixture was measured and plotted against the percentage of drug fraction (w/w%) in the physical mixture (Example plots are shown in Fig. 3 and plots for the remaining drugs are shown in the ESI Fig. S3 and S4†). The apparent drug solubility in PLGA polymer was obtained from the line intercept with the *X*-axis. As shown in Table 3, the drugs can be ranked according to their solid-state solubility in PLGA.

**Table 3:** Experimental drug-polymer solid state solubility measured by zero enthalpy DSC method

Drug	Log *P*	Solubility of drug in PLGA (%W/W)	Error of fit (%W/W)	Solubility of drug in PLGA (mol per 100 g)
KPN	3.29	1.34	0.96	0.53
IND	4.25	6.24	0.58	1.74
SFN	4.34	7.32	0.60	1.58
CFZ	7.39	9.65	0.21	2.04

If solubilities are quantified in weight/weight terms the rank order of solubilities is: CFZ > SFN > IND > KPN. But if the rank order is in molar terms the order of IND and SFN is reversed and the ratio of solubilities between the drugs is reduced. For example, the weight ratio of solubilities of KPN and CFZ is 1 : 7.2, but the molecular ratio is 1 : 3.8. This demonstrates the importance of using molecular concentration units if the concern is to understand mechanisms at the molecular level.

The solid-state solubility data are roughly in line with the log *P* values suggesting that greater hydrophobicity results in greater solid solubility in solid PLGA. Therefore, based on the assumption adopted in the literature that using the solid solubility of CFZ in PLGA, this drug would be the drug with the greatest loading capacity within PLGA NPs.^51–53^ This will be tested and discussed in the following sections.

### 3.2. The effects of drug/polymer solubilities in solvent/antisolvents on precipitation dynamics

It is believed that blank PLGA NPs prepared by nanoprecipitation are formed in three stages, involving NPs nucleation, growth through aggregation, and eventually the formation of kinetically locked nanoparticles after a characteristic aggregation time scale^4,18,54–57^ However, the situation may be complicated by the presence of drug which could interfere with nucleation and growth and may itself form crystals if supersaturation conditions occur. In order to gain an insight into the possible mechanisms involved during the mixing process for solvent and anti-solvent and the role of drug it is necessary to consider both the kinetic and thermodynamic factors involved. In the mixing process for the formation of nanoparticles, the drop enters the solution and then must be rapidly mixed so it can be assumed that if the total time for addition is 1 minute as in the experiments reported here, the rate of concentration change is 1/60 of the total mass of drug per second in a solution of 4 mL of water plus 1/60 of a millilitre of ACN solution per second. Table 4 shows the timescales needed to reach the saturation concentration of the drug in water for the different drugs. For CFZ and SFN the timescale is relatively long and by that time the ACN content is high. On the other hand, this is not the case for IND and KPN.

**Table 4:** Calculated time scale, ACN content and total liquid volume at which added drug reaches its saturation concentration in water

Drug	Solubility in water (μg mL^−1^)	Total mass of drug in ACN (μg)	Calculated time of drug concentration reaching saturation level in water (s)	ACN added volume at which water saturation time is reached (mL)	Total volume (mL)
IND	16.64 ± 4.78	568	7.45	0.12	4.12
KPN^a^	202 ± 34.11	15 760	3.12	0.05	4.05
SFN	0.18 ± 0.01	1.23	41.18	0.69	4.69
CFZ	0.01 ± 0.01	0.668	49.25	0.82	4.82

a Data for KPN at 0.8 DOS.

A probable course of events is that: as the drop is added there is rapid precipitation of PLGA and, in the case of CFZ and SFN, no precipitation, or very transient formation of precipitate followed by redissolution. In the case of IND and KPN there is much more likely to be precipitation followed by slower redissolution, as the system will require more ACN to ensure that the system is undersaturated. A note of caution is needed here as the difference in solubility of these two drugs in water and ACN is very large, so it is possible that low levels of solvent may be sufficient to cause dissolution. There is also the possibility that the precipitation of these two drugs is slow, and that the solution may remain supersaturated at the early stage of addition. Alternatively, redissolution may be slow so that the solution is undersaturated.

The entrapment of drug by the polymer may occur at the polymer precipitation stage where drug is held by a section of polymer chain and is carried into the nanoparticle. Another mechanism might be that drug is absorbed on the growing nanoparticle and remains during growth, or drug is absorbed on the fully formed nanoparticle.

In all the cases, the drug interacts with the polymer either in solution or by absorption on a growing nanoparticle or on the mature particle. These events can be treated by assuming a simple reaction mechanism and that equilibrium or pseudo-equilibrium is reached. Thus, the reaction may be characterised by the eqn (6).

6 *D* + *P* ↔ DP

where *D* is the drug interacting with polymer *P* to form a complex DP. The polymer is a long chain consisting of monomeric subunits. If the polymer consists of *n* oligomeric units of length *m* monomeric subunits and one drug molecule is assumed to react with one oligomeric unit to form a complex DP*, the formation of the complex can be rewritten as

7 *D* + P* ↔ DP*

where P* is an oligomeric subunit and the concentration of the uncomplexed oligomeric subunits is expressed as [P*].

The equilibrium may be described by an equilibrium constant *K*.8
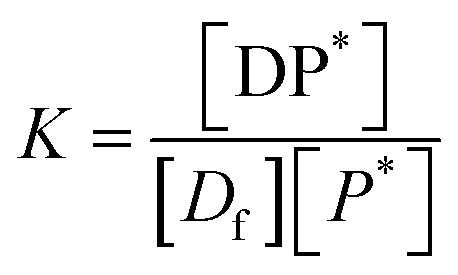
where [*D*_f_] is the concentration of uncomplexed drug and [DP*] is the concentration of the complex.

If [*D*_0_] is the total concentration of the drug in the system, then

8a [*D*_f_] = [*D*_0_] − [DP*]

and8b
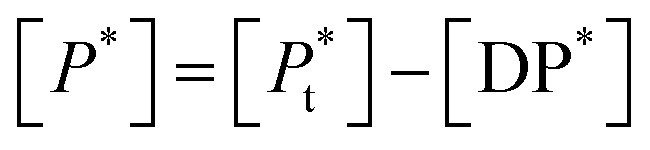
where 
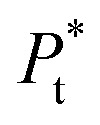
 is the total concentration of the oligomers.

P* = *P/n*, but value of *n* is not known, and may be variable, so eqn (8) can be rewritten in terms of a pseudo constant K* such that K* = *K*/*n* and P* = *P*/*n*. Thus,9
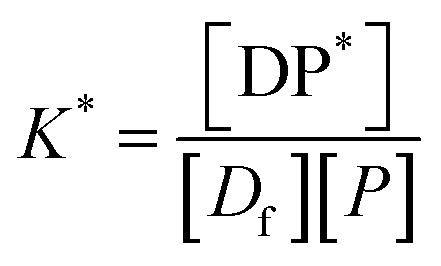


From eqn (8a) and (8b):

Since we can write P* = *P*/*n*, then 
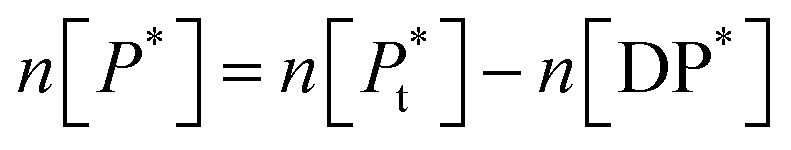
 thus [*P*] = [*P*_t_] − *n*[DP*].

Substituting in eqn (9) and rearranging we obtain:10
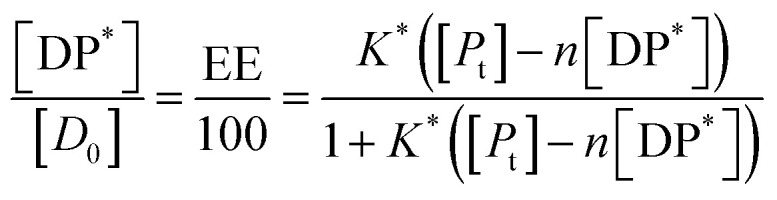
where *P*_t_ is the total amount of polymer present expressed in mols of monomer units (*i.e.* the average molecular weight of the two component subunits) and *D*_0_ total number of mols of drug present and EE is the entrapment efficiency expressed in molar terms.

In the limit *n*[DP*] ≪ [*P*_t_], eqn (10) becomes11
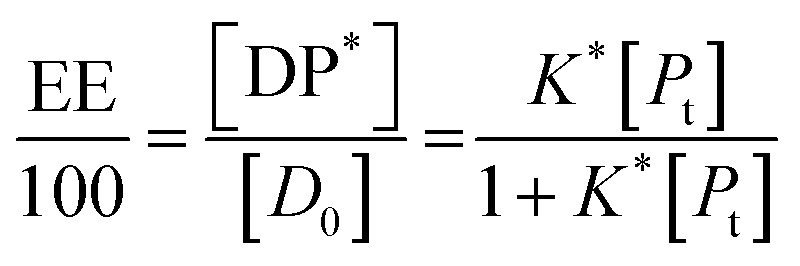


From eqn (9) and the relationship [*P*] = [*P*_t_] − *n*[DP*] it follows that12
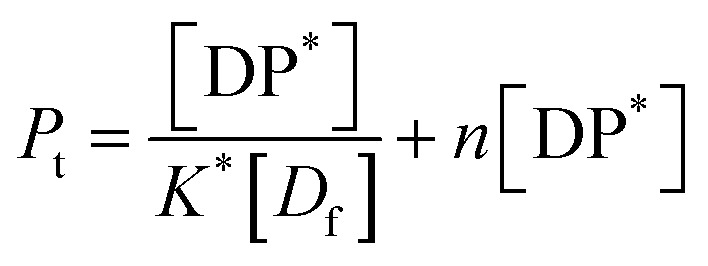


From which13
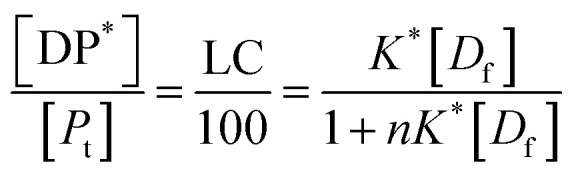


Eqn (13) is equivalent to the Langmuir absorption isotherm and the left-hand side of the equation is a form of the loading capacity expressed in molar units.

If eqn (11) holds, then since by definition,14
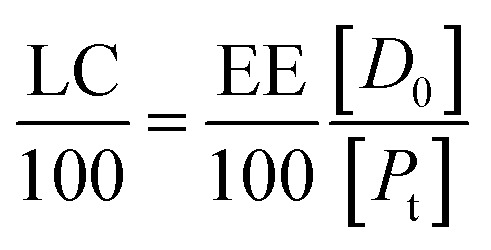


It follows that15
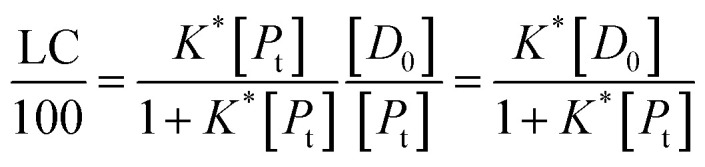


If the PLGA concentration is kept constant LC is a linear function of *D*_0_ and if *D*_0_ is constant LC is inversely proportional to *P*_t_. In the limit that K*P_t_ is very small and *D*_0_ is constant then LC will remain invariant with *P*_t_. This corresponds to a situation in which either K* or *P*_t_, or both, are very small, and the polymer has reached the limit of its ability to entrap drug.

Testing the model discussed above encounters a number of significant practical problems which must be addressed. The correct value for the mass of PLGA interacting with the drug is uncertain, the measured equilibrium value of the mass of PLGA soluble in the ACN/water mixture is 106 μg mL^−1^ but it not certain how much of the polymer is dissolved at the time of measurement, as the system is not necessarily at equilibrium for the polymer solution. The values given in Table 5 are based on the assumption that none of the polymer is dissolved. The assumption that the polymer is at equilibrium solubility in fact changes the constant by a relatively small value of the order of the errors of measurement. Data can be found in the ESI (Fig. S4, S5 and Table S1).†

**Table 5:** Results of curve fits of the EE data from Fig. 4 to eqn (11)

Drug	DOS	Initial concentration in 20% ACN/water (μmol mL^−1^)	K* (mL μmol^−1^)	Estimated error of fit	100K*D_0_
CFZ	0.8	2.822 × 10^−4^	1.48 × 10^−1^	4.90 × 10^−3^	0.0042^a^
SFN	0.8	5.284 × 10^−4^	4.90 × 10^−2^	4.60 × 10^−3^	0.0026^a^
IND	0.8	0.3175	1.20 × 10^−2^	9.10 × 10^−4^	0.38
KPN	0.2	3.099	2.00 × 10^−3^	1.94 × 10^−5^	0.62
KPN	0.4	6.197	2.36 × 10^−3^	1.50 × 10^−4^	1.46
KPN	0.8	12.39	4.08 × 10^−3^	1.20 × 10^−4^	5.055

a These values are not constant but are taken in the mid PLGA range to illustrate the order of magnitude between these and the other drugs. Where K* is the equilibrium pseudo constant of drug–polymer interaction and 100K*D represents loading capacity expressed in molar terms.

Another problem with measurement using the indirect method, is that, where the drug is highly soluble, and the entrapment efficiency is low, the calculation of the amount of drug entrapped by polymer is the difference of two large quantities. For example, in the case of KPN at 0.8 DOS and an EE of 5%, a 1% error on the measurement of the free drug will result in an 18% error in the calculation of [DP*]. As an example of the effect of this is that the probable error of free drug concentration for the 0.8 KPN experiment is around 2.5%. This is because the value of the free drug, estimated at zero PLGA concentration by extrapolation of the free drug concentrations is less than the measured value of the solubility of the drug by 2.5%. The resulting value of K* reduces from 5.6 × 10^−3^ mL μmol^−1^ to 4.08 × 10^−3^ mL μmol^−1^ if the extrapolated value of free drug concentration is used. With other levels of saturation of KPN the extrapolated values of *D*_0_ and the concentration of initial mass of drug are very close.

The fits to the data for IND and KPN are very close to linear. In this case eqn (11) reduces to:16
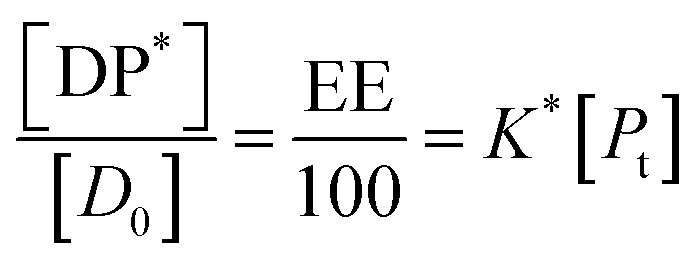


And therefore:17
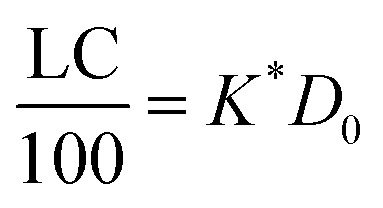


Thus, loading capacity, expressed in molar terms, is a constant of value 100 × K*[*D*_0_], these are listed in Table 5.

**Fig. 4:**
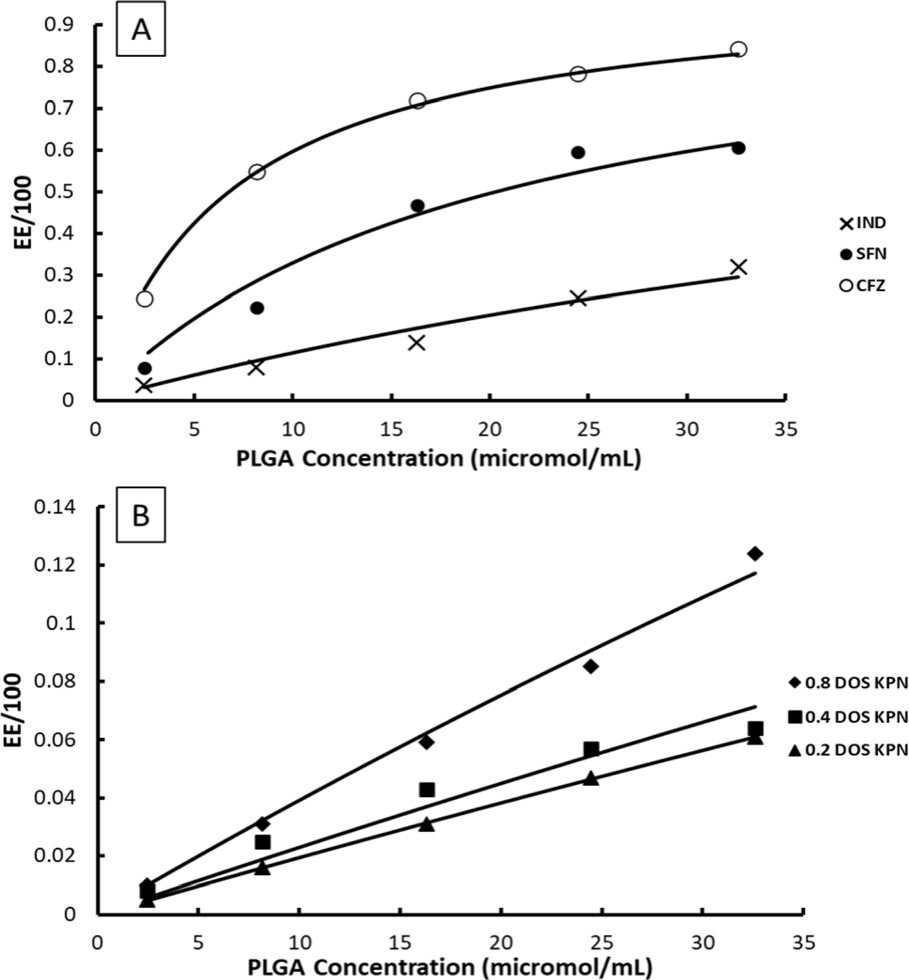
Examples of fits of eqn (11) to experimentally measured EE data, (A) CFZ, IND and SFN, (B) KPN at different degrees of saturation.

Example fits to eqn (11) are shown in Fig. 4 and data is summarised in Table 5.

The values of EE for SFN and CFZ and the values of 100K*D_0_ vary with PLGA concentration so those shown in Table 5 are chosen from the mid polymer concentration range. Whilst the constant K* is high for SFN and CFZ and consequently the EE is high, the loading capacity is 2 to 3 orders of magnitude lower than for IND and KPN. This is a consequence of the low values of *D*_0_ due to low solubility of SFN and CFZ.

The question arises as to whether the mechanism of drug entrapment is by surface absorption or by inclusion in the bulk of the nanoparticle following interaction with the polymer, as both mechanisms would give the same equations. Measurement of the surface absorption by addition of drug to naked nanoparticles indicates that a surface absorption mechanism is credible. Table 6 shows the EE% from precipitation experiments compared to those from surface absorption experiments.

**Table 6:** Comparison of the EE% values of the KPN loaded nanoparticles prepared by precipitation and surface absorption method

Final KPN DOS	Final PLGA DOS	EE (%) from nanoprecipitation study	EE (%) due to surface adsorption
0.2	5	1.6 ± 0.2	2.2 ± 0.1
0.4		2.5 ± 0.2	2.5 ± 0.04

These data show that the similarity in the values of EE for both precipitation and absorption show that surface absorption model is credible but does not exclude a role for incorporation of drug since penetration of the drug into the nanoparticle is not impossible.

The measurement of particle size as function of PLGA concentration shows that the size of the particles increases as PLGA concentration increases (Table 7). The total surface area for a total mass of particles *M*_t_, density *ρ* and radius *r* is given by 3*M*_t_/*rρ.*

**Table 7:** Variation of particle size and total surface area of blank PLGA NPs as a function of PLGA concentration

PLGA concentration in ACN (mg mL^−1^)	PLGA DOS in 20% ACN/MQW	Z-AVG (nm)	3*M*_t_/*rρ* (cm^2^ mL^−1^)	PDI
1	1.9	88.3 ± 3	109	0.2 ± 0.01
5	9.4	143 ± 5	335	0.1 ± 0.02
10	18.9	167 ± 3	574	0.08 ± 0.01

From Table 7, it is apparent that as the PLGA concentration increases the particle surface area to mass ratio increases but at a rate less than the increase in PLGA concentration suggesting that surface absorption may not be the mechanism of drug entrapment.

TEM images of the blank PLGA NPs prepared using DOS 9.4 are shown in the ESI (Fig. S6†). The NPs are spherical in shape. These nanoparticles are smaller in size than measured using DLS analysis, which is likely to be caused by the shrinkage during drying and staining of the TEM samples preparation.

An issue with KPN is the variation of K* with degree of supersaturation. Whilst the values for the 0.2 and 0.4 DOS are close, the value for 0.8 DOS is significantly larger. In order to examine the possible reasons for it, it is useful to consider in more detail how the relationship between LC and EE varies with drug concentration at constant polymer concentration and with polymer concentration at constant drug concentration. From eqn (14), if the PLGA concentration is kept constant, LC is a linear function of *D*_0_ and, if *D*_0_ is constant, LC is inversely proportional to *P*_t_. In the limit that K*P_t_ is very small then the polymer will become saturated with the drug and LC will be constant as drug concentration rises. In general, if the drug concentration can reach a high enough level, then saturation will occur. It should be recognised that if the solubility limit of the drug is exceeded, and supersaturation has not occurred, then the concentration of free drug in solution will remain constant and LC will also remain constant even though the saturation limit of the polymer is not reached. Eqn (13) predicts that under conditions where the polymer can accommodate no more drug molecules, the EE declines proportionally to 1/*D*_0_. It should be noted that eqn (13) simply depends on the definitions EE and LC not on any particular model.

If the free energy of the entrapment process of the drug by polymer is great enough, then drug may be absorbed from solution thus causing more drug to dissolve from the crystalline material allowing LC to increase. The reverse process is not possible that is: if the solution is saturated with drug, then the crystals cannot absorb drug from solution or from the polymer drug complex.

In the case of a constant value of total drug concentration, the situation is somewhat simpler. If the saturation limit of the initial polymer amount is reached, then LC will remain constant and will only decline if the increase in polymer amount is sufficient to reach a point where the saturation conditions do not apply. In this case LC will decrease continuously with polymer amount increase. At low polymer amount, EE will increase, and at high enough polymer amount, it will asymptotically approach 100% whilst LC will asymptotically approach zero.

From the foregoing considerations the apparent variation of K* with KPN supersaturation cannot be explained simply on the basis of straightforward concentration effect. The results are consistent with a difference in the ability of the nanoparticles to absorb KPN, implying that in the preparation stage the drug concentration has a strong effect on the nature of the nanoparticles precipitated. This means that measured values of K* may only be valid for a particular set of preparation conditions.

One of the major problems in choosing a system for production of drug loaded nanoparticles is that the correlation between the physical properties of the drug and polymer and the final behaviour of the system is poor. Table 8 lists some commonly used physical parameters in prediction of behaviour and the observed outcomes of drug loading. In this study, drug solubility in the solvent/antisolvent mixture has been shown to have a substantial effect on both EE and LC.

**Table 8:** Parameters of the drug entrapment process compared to often used physical parameters in literature for drug loading behaviour prediction. Data for EE and LC taken at [*P*] = 16.3 μmoL mL^−1^

Drug	DOS	Initial concentration (μmol mL^−1^)	Log *P*	Solubility in solid PLGA (mol per 100 g)	Solubility in 20%ACN/water (μmol ml^−1^)	K* (mL μmol^−1^)	EE (% w/w)	LC (mol g^−1^)
CFZ	0.8	2.822 × 10^−4^	7.39	2.04	3.53 × 10^−4^	1.48 × 10^−1^	71.8	1.3 × 10^−3^ ± 1 × 10^−4^
SFN	0.8	5.284 × 10^−4^	4.34	1.58	6.60 × 10^−4^	4.90 × 10^−2^	46.9	1.4 × 10^−4^ ± 2 × 10^−5^
IND	0.8	0.3175	4.25	1.74	3.69 × 10^−1^	1.20 × 10^−2^	13.9	0.57 ± 0.03
KPN	0.2	3.099	3.29	0.53	15.9	2.00 × 10^−3^	3.1	1.65 ± 0.07
KPN	0.4	6.197	3.29	0.53	15.9	2.36 × 10^−3^	4.3	1.46 ± 0.07
KPN	0.8	12.39	3.29	0.53	15.9	4.08 × 10^−3^	5.9	4.49 ± 0.03

Clearly the data is limited in extent but no simple correlation between physical parameters and either K* or EE or LC is apparent. K* varies over 2 orders of magnitude and LC by 4 orders of magnitude, this is largely due to the variation of the solubility of the drugs in ACN/water mixture. This effect is particularly notable in the comparative behaviour of IND and SFN both have similar values of Log *P*, solubility in PLGA and K* but differ in 3 orders of magnitude in LC. This is due to their differences in solubility in the ACN/water mixture.

## 4. Conclusion

To understand the mechanisms involved in the use of the nanoprecipitation method for the production of PLGA nanoparticles in the presence of a drug requires consideration of the both the kinetic and thermodynamic factors involved. During the process in which a solution of drug and polymer is mixed with water, both precipitation of the polymer nanoparticle and drug can occur.

Both of these depend on the solubility of the components, in the case of PLGA it can be assumed that precipitation is rapid, but in the case of the drug it will depend on the solubility of the drug in the solvent system, which changes as drug/polymer solution is added. In the case of the drug, both the solubility in the changing solvent system and the kinetics of crystallisation and redissolution will play a role.

Even so a simple equilibrium type model can be used to fit the data for the variation of EE with PLGA concentration. Such a model requires units of quantity to be molecularly based, rather than mass based, as is usual in work of this kind in the existing literature. We stress that it is important that, if mechanisms are to be understood in molecular terms, these units must be used.

The entrapment of drug by polymer can be characterised by pseudo constant K*. The largest value of this parameter is observed for CFZ and SFN. However, the values of LC are very low. Even though EE for these compounds is high. This arises because the solubilities of these drugs are very low and therefore the amount of material available to load into the polymer is small. In contrast, the values of K* and EE are small for IND and KPN, but LC is larger than for the other drugs, simply due to the large amounts of drug available (due to their higher solubility in the solvent mixes). The indications of these results are that for SFN and CFZ optimum efficiency would be to minimise the amount of polymer, to ensure maximum loading, and the reverse case for KPN and IND to maximise the removal of drug from solution. The value of K* itself is not an indicator of the outcome of a particular loading experiment, which is very much dependent on the amount of drug available to interact with the polymer. K* therefore, is a means of calculating the outcome under a particular set of conditions.

In the literature there has been interest in predicting dug polymer interactions by consideration of various physical parameters, such as log *P*, the solubility of the drug in pure polymer and the solubility of the drug in the solvent systems.

The solubilities of the drug in the solvent system and in pure polymer have been measured experimentally in this work. The latter measurement was made by the well-known zero enthalpy method, but we stress the outcome is not a straightforward measure of solubility but a rough approximation. For the range of systems tested here no clear coherent correlation between these parameters and K* was observed.

In summary:

We have developed a method for systematic measurement of the affinity of drug for PLGA nanoparticles using a constant degree of saturation to maintain an approximately constant thermodynamic activity.

We have derived a relationship that expresses the affinity of the drug as a single parameter, which is pseudo equilibrium constant. We also discuss the limitations of the measurement of solubility of the drug in polymer by the zero-enthalpy method and derive a general expression for the enthalpy values obtained in the case where a fraction of drug dissolves in the heating stage and more dissolves at the temperature of the fusion of the crystalline drug.

## Conflicts of interest

There are no conflicts to declare.

## Supplementary Material

NA-006-D4NA00087K-s001
