# Supplementary material for: Using a systematic and quantitative approach to generate new insights into drug loading of PLGA nanoparticles using nanoprecipitation
Source: Nanoscale Adv. 2024 May 8;6(12):3188–98. doi: 10.1039/d4na00087k (PMC11166107; doi:10.1039/d4na00087k)
Supplement: NA-006-D4NA00087K-s001 [file NA-006-D4NA00087K-s001.pdf]

Electronic Supplementary Material (ESI) for Nanoscale Advances.
This journal is © The Royal Society of Chemistry 2024

Supplementary information

Received 00th January 2024,

Using a systematic and quantitative approach to generate new insights into drug loading of PLGA nanoparticles using nanoprecipitation

Accepted 00th January 20xx

DOI: 10.1039/x0xx00000x

Sherif I. Hamdallah[¤a,b], Randa Zoqlam[¤c], Bin Yang[d], Andrew Campbell[d], Rebecca Booth[e], Jonathan Booth[e], Peter Belton[f], Sheng Qi[*a].

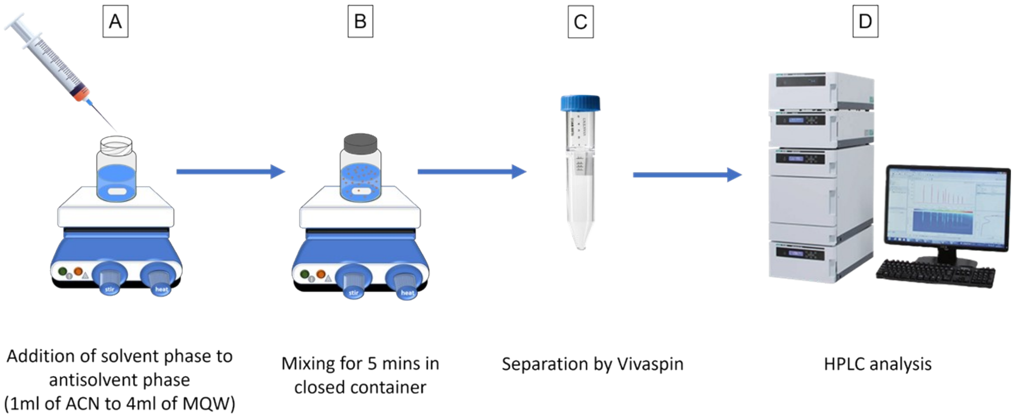

Figure S1: Schematic presentation of the procedure used to study drug-PLGA interaction (represented by loading capacity) in the 20% ACN/MQW solvent/antisolvent mixture: (A-B) demonstrates the preparation of the drug loaded NPs, (C) separation of the free drug using Vivaspin® and (D) quantification of the drug loading using HPLC.

The derivation of the enthalpy of fusion of a drug in contact with polymer:

The system contains a polymer rich phase P_1 and drug rich phase P_2. The solubility of the drug in polymer is defined as:

[a.] *School of Pharmacy, University of East Anglia, Norwich NR4 7TJ, UK. E-mail: S.hamdallah@uea.ac.uk, *Sheng.qi@uea.ac.uk*
[b.] *Department of Pharmaceutics, Faculty of Pharmacy, Alexandria University, Alexandria, Egypt.*
[c.] *School of pharmacy, university college London, London WC1E 6BT, UK. E-mail: r.zoqlam@ucl.ac.uk.*
[d.] *Advanced Drug Delivery, Pharmaceutical Sciences, The discovery Center (DISC), 1 Francis Crick Avenue, Cambridge, CB2 0AA, UK. E-mail: bin.yang4@astrazeneca.com, Andrew.Campbell@astrazeneca.com*
[e.] *New Modalities and Parenteral Development, Pharmaceutical Technology & Development, Operations, AstraZeneca, Macclesfield SK10 2NA, U.K. E-mail: Rebecca.J.Booth@astrazeneca.com, Jonathan.Booth@astrazeneca.com.*
[f.] *School of Chemistry, University of East Anglia, Norwich NR4 7TJ, UK. E-mail: p.belton@uea.ac.uk*
Electronic Supplementary Information (ESI) available: [details of any supplementary information available should be included here]. See DOI: 10.1039/x0xx00000x
[¤] Co-first authorship with equal contribution.

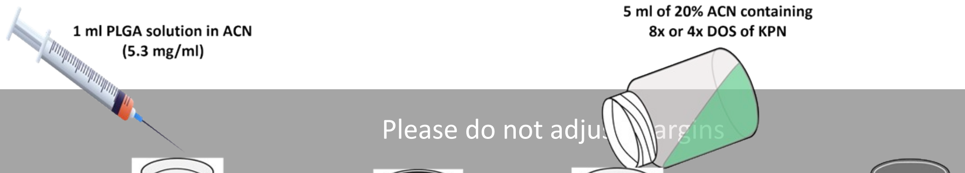

$$K_D = \frac{M_{D1}}{M_{P1}} \quad \text{Eq. (1)}$$

Where, M_{D1} is the mass of drug dissolved in polymer at equilibrium and M_{P1} is the mass of polymer in phase 1 (P_1).

Similarly, the solubility of polymer in molten drug is defined as:

$$K_P = \frac{M_{P2}}{M_{D2}} \quad \text{Eq. (2)}$$

Where, the subscripts refer to polymer and drug in the drug rich phase.

The mass balance of the drugs and polymer is $M_D= M_{D1}+ M_{D2}$ and $M_P= M_{P1}+ M_{P2}$, respectively, when M_D is below the mass of drug soluble in polymer, the heating process may cause dissolution of the drug before the melting point of the drug crystals is reached. This will depend on the kinetics of dissolution which will be affected by factors such as heating rate and the intimacy of the drug/polymer mixture. If some fraction, F_1, of the soluble mass is dissolved before the melting point of the drug is reached then:

$$F_1 M_{D1} = F_1 K_D M_{P1} \quad \text{Eq. (3)}$$

The fraction not dissolved is F_2, and thus $F_1+F_2=1$. The solubility of the polymer in drug is assumed to be small and will only occur when the drug to polymer ratio is very high according to Qi, S. *et al.* [1] Therefore, M_{P2} is small hence $M_P \approx M_{P1}$ and $M_D \approx M_{D2}$, and consequently $M_D K_P = M_{P2}$. The amount of crystalline drug remaining above the saturation limit is:

$$M_D - M_{D1} = M_D - F_1 K_D M_P \quad \text{Eq. (4)}$$

When drug dissolves in polymer there will be a heat of dissolution, H_{DS}, which, in general, will be exothermic. The heat evolved in dissolution will be:

$$H_{DS} = F_2 K_D M_P \quad \text{Eq. (5)}$$

If the heat of fusion is H_F, then the heat change on melting will be:

$$H_F = [M_D - F_1 K_D M_P] \quad \text{Eq. (6)}$$

Similarly, the heat of dissolution of the polymer in drug will be $H_P K_P M_D$. where, H_p is the heat of dissolution of the polymer in the molten drug. The total heat change on melting will be:

$$H_T = H_F[M_D - F_1 K_D M_P] + H_{DS} F_2 K_D M_P + H_P K_P M_D \quad \text{Eq. (7)}$$

It is important to note that if drug dissolves before the melting point the total mass of drug available to melt will be reduced and if saturation is not reached before melting then the heat of dissolution of the drug will contribute to the observed heat change on melting. From Eq. 7, the heat released per unit mass of drug plus polymer is:

$$H_{TM} = H_F[X_D - F_1 K_D(1 - X_D)] + H_{DS} F_2 K_D(1 - X_D) + H_P K_P M_D \quad \text{Eq. (8)}$$

Where X_D is the weight fraction of drug in the system.
Eq. 8 is linear in X_D in the region where $H_P K_P X_D << H_F[X_D - F_1 K_D(1-X_D)] + H_{DS} F_2 K_D(1-X_D)$, which is the case for most levels of drug to polymer ratio except when the drug to polymer ratio is very high. When $F_1= 0$: i.e. no dissolution before melting, then

$$H_T = X_D[H_F - H_{DS} K_D] + K_D H_{DS} + H_P K_P M_D \quad \text{Eq. (9)}$$

Since the term $H_P K_P X_D$ is small and can only become significant when X_D>1, it may be ignored at low weight fractions of polymer. Hence, when F_1= 1: i.e. dissolution saturates the polymer before melting,

$$H_T = X_D[H_F + H_F K_D] - K_D H_F \quad \text{Eq. (10)}$$

From Eq. 9 when H_T =0,

$$X_D = K_D H_{DS}/[H_{DS} K_D - H_F] \quad \text{Eq. (11)}$$

This journal is © The Royal Society of Chemistry 20xx

Please do not adjust margins

From Eq. 10 when H_T =0,

$$X_D = K_D H_F/[H_F(1 + K_D)] \quad \text{Eq. (12)}$$

In Eq. 9, H_F and H_D will typically have opposite signs and since $|H_F| > |H_{DS}K_D|$, X_D is positive. In Eq. 10 if $K_D << 1$ then $X_D = K_D$ and the intercept on the *X* axis is the solubility of the drug in polymer.

It is not usually possible to determine with certainty what the value of F_1 is as at low drug levels as the heat change on melting may be small and partly compensated by the heat of dissolution of the drug in polymer, so that no heat change is detected.

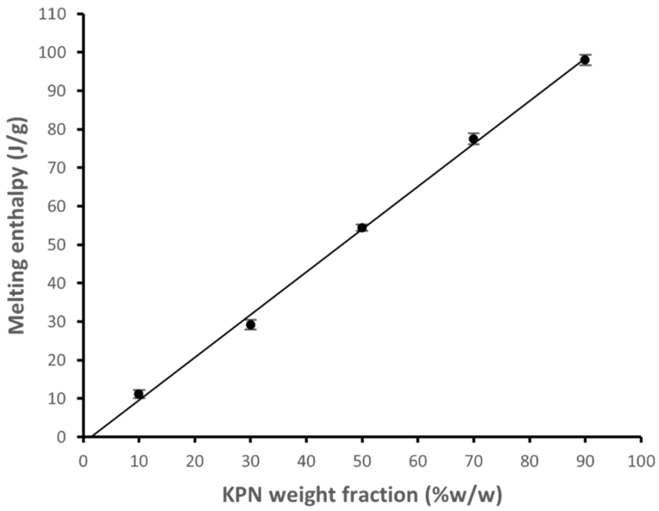

Figure S3: Plots of melting enthalpy of KPN with PLGA physical mixtures.

Please do not adjust margins

In the case of clofazimine there maybe evidence of the dissolution of polymer in drug as shown in Figs S4 and S5

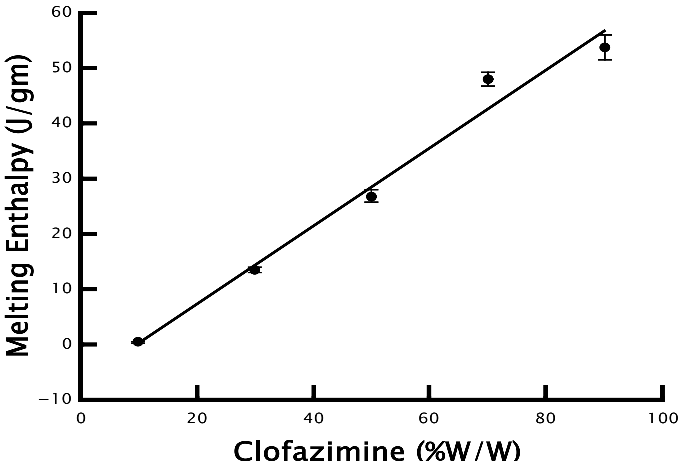

Figure S4: Straight line fit of CFZ enthalpy of melting in contact with PLGA with 5-point data fit. Note the point at 80% PLGA deviates from the fit by more than standard deviation of measurement.

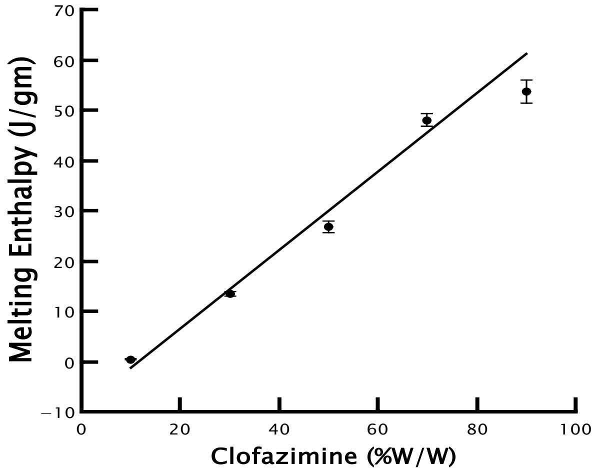

Figure S5: Straight line fit of CFZ enthalpy of melting in contact with PLGA with 4-point data fit excluding the 90% data point. 90% PLGA deviates from the fit by more than standard deviation of measurement. The deviation of the 90% data point in Figure S4 is consistent with the effect of polymer dissolution in the molten drug as discussed above.

 This journal is © The Royal Society of Chemistry 20xx

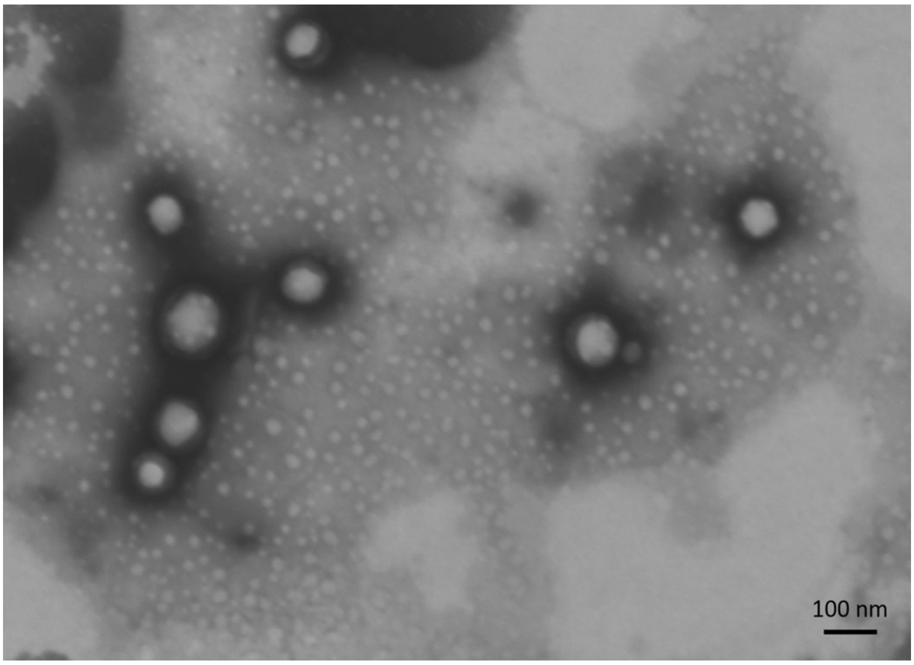

Figure S6: TEM images of blank PLGA NPs prepared at 9.4x DOS of PLGA.

Table S1: Values of K* when assuming that (a), that the solubilities of the PLGA in the solvent mixture is zero and (b) when it is the equilibrium measured solubility.

Drug	DOS	K* (a) (/(ml/µmol)	estimated error of fit	K*(b)	estimated error of fit
**CFZ**	0.8	1.48×10^{-1}	4.90×10^{-3}	1.92×10^{-1}	2.09×10^{-2}
**SFN**	0.8	4.90×10^{-2}	4.60×10^{-3}	5.57×10^{-2}	4.47×10^{-3}
**IND**	0.8	1.20×10^{-2}	9.10×10^{-4}	1.40×10^{-2}	9.5×10^{-4}
**KPN**	0.2	2.00×10^{-3}	1.94×10^{-5}	2.60×10^{-3}	3.5×10^{-6}
**KPN**	0.4	2.36×10^{-3}	1.50×10^{-4}	2.52×10^{-3}	2.13×10^{-4}
**KPN**	0.8	4.08×10^{-3}	1.20×10^{-4}	4.43×10^{-3}	1.51×10^{-4}

Please do not adjust margins

References

1 S. Qi, P. Belton, K. Nollenberger, N. Clayden, M. Reading and D. Q. M. Craig, *Pharm. Res.*, 2010, **27**, 1869–1883.

This journal is © The Royal Society of Chemistry 20xx

Please do not adjust margins
